# Surface Modification of Biomass with Di-(2-Ethylhexyl)phosphoric Acid and Its Use for Vanadium Adsorption

**DOI:** 10.3390/ma15207300

**Published:** 2022-10-19

**Authors:** Zhekun Yu, Yong Fan, Tao Liu, Yimin Zhang, Pengcheng Hu

**Affiliations:** 1 School of Resource and Environmental Engineering, Wuhan University of Science and Technology, Wuhan 430081, China; 2 State Environmental Protection Key Laboratory of Mineral Metallurgical Resources Utilization and Pollution Control, Wuhan University of Science and Technology, Wuhan 430081, China; 3 Collaborative Innovation Center of Strategic Vanadium Resources Utilization, Wuhan 430081, China; 4 Hubei Provincial Engineering Technology Research Center of High Efficient Cleaning Utilization for Shale Vanadium Resource, Wuhan University of Science and Technology, Wuhan 430081, China

**Keywords:** vanadium, biomass, adsorption capacity, di-(2-ethylhexyl)phosphoric acid

## Abstract

The method of carbonizing biomass using di-(2-Ethylhexyl) phosphoric acid and tributyl phosphate impregnation (SICB) was studied in this research. SICB combines the benefits of an extractant and an ion exchange resin. The adsorption and desorption properties of vanadium were investigated, and the adsorption mechanism was analyzed. The results showed that the carrier was first prepared at a temperature of 1073.15 K using sawdust as a biomass substitute and then cooled to room temperature. The best adsorption performance was obtained by impregnating the carriers with di-(2-Ethylhexyl) phosphoric acid and tributyl phosphate for 60 min. The vanadium adsorption rate of 98.12% was achieved using the biomass at an initial V(IV) solution concentration of 1.1 g/L, a pH value of 1.6, and a solid-to-liquid ratio of 1:20 g·mL for 24 h. Using 25 wt.% sulfuric acid solution as desorbent, the desorption rate of vanadium was as high as 98.36%. The analysis showed that the adsorption of vanadium by SICB was chemisorption, and the adsorption process was more consistent with the proposed second-order kinetic equation. Therefore, SICB has high selectivity and high saturation capacity because of the mesopores and micropores produced by carbonization.

## 1. Introduction

Vanadium is a strategically important rare metal with a high melting point, high hardness, low density, good corrosion resistance, and stable nuclear physical properties. It is used in metallurgy, aerospace, the nuclear industry, hydrogen storage materials, and may be used in other fields such as chemistry, batteries, pigments, glass, optics, and pharmaceuticals [1]. China’s vanadium resources were mainly derived from vanadium shale and vanadium-titanium magnetite, of which vanadium shale was widely distributed, with proven reserves of 61.88 billion tonnes. Due to the proven reserves of 77.075 million tonnes of vanadium pentoxide in vanadium shale, the extraction of vanadium from vanadium shale was an important development direction for China’s vanadium industry [2].

V(III) was the predominant form of vanadium found in vanadium shale. The process of recovering vanadium from vanadium shalecaused oxidation of insoluble V(III) to acid-soluble V(IV) compounds and/or water-soluble V(V) compounds, which were then dissolved by acid leaching and/or water leaching [3,4]. However, many impurities were usually dissolved in the acid leach solution together with vanadium, which made the separation of vanadium and impurities in the acid leach solution a necessary process [5,6]. In recent years, many researchers have focused on the purification and enrichment of vanadium from acid leach solutions. Vanadium can be separated and enriched from acid leaching solutions through solvent extraction processes and ion exchange processes. The most common process for the purification and enrichment of vanadium is solvent extraction, with organic phases such as D2EHPA, TBP, LIX63, PC88A, N235, etc. [7,8,9,10]. The process is highly selective and has a high treatment capacity [11]. The ion exchange process is also used for the purification and enrichment of vanadium, which is highly selective, easy to operate, and environmentally friendly. Some studies have used [12,13,14,15] the combination of ion exchange resin and solvent extraction to prepare for impregnating resin, which combines the advantages of ion exchange resin and solvent extraction. However, due to the low saturation adsorption capacity of impregnating resin, the utilization rate of extractant was low. Therefore, it is important to prepare a high saturation adsorption capacity and high solvent utilization rate of the adsorbent material. Biomass comes from a wide range of sources and is a green, biodegradable, and sustainable material. The biomass chosen for this paper is domestic waste containing wood fibers. It is readily available, plentiful, inexpensive, and can be reused as waste. The material made by high-temperature carbonization of biomass is a high-quality adsorbent material with a loose and large pore structure and large surface area [16,17]. The carbonized biomass itself has some physical adsorption effect, but is not selective and has a poor adsorption effect on vanadium. Thus new adsorption sites need to be added, and impregnation is one of them [18,19]. The carbonized biomass was used as a carrier and the adsorbent was loaded onto the biomass by impregnation to produce a new adsorbent material. The adsorption material was made selective and then went on to adsorb specific metal ions. In recent years, many researchers have investigated the adsorption of metal ions on carbonized biomass. For example, biomass such as sesame straw biochar, agricultural waste, hazelnut shells, fruit biomass, banana peels, and orange peels were used as carriers of adsorbent materials to adsorb heavy metal ions from wastewater [20,21,22,23,24,25]. The use of biomass as a matrix for adsorbent materials has become a viable process [26]. Therefore, it is expected that a sorbent material can be prepared by using biomass as a substrate and impregnating the extractant to solve the problems of low saturation sorption capacity and low solvent utilization of solvent-impregnated resin for V extraction.

This study aimed to solve the problems of low saturation adsorption capacity and low solvent utilization of solvent-impregnated resin in order to prepare a new adsorbent material for solvent-impregnated carbonized biomass (SICB) and use this adsorbent material to achieve efficient extraction of vanadium. The optimal preparation and adsorption conditions of SICB were investigated, and the adsorption mechanism of vanadium was analyzed using Isotherm, Kinetic, and Elovich kinetic models. The feasibility and mechanism of treating vanadium solutions by biomass solvent impregnation were investigated to provide a new process for the purification and enrichment of vanadium shale leachate.

## 2. Materials and Experiment Methods

### 2.1. Materials

Di-(2-Ethylhexyl) phosphoric acid (D2EHPA), tributyl phosphate (TBP), and anhydrous ethanol were purchased from China National Pharmaceutical Group Chemical Reagent Co., Beijing, China. The vanadium solution containing 1.1 g/L V(IV) was prepared by dissolving VOSO_4_·xH_2_O in deionized water. The pH of the solution was adjusted using sulfuric acid and A.R. grade sodium hydroxide solution.

### 2.2. Experimental

#### 2.2.1. Preparation of SICB

The biomass (poplar sawdust) was dried in a vacuum dryer to remove moisture. The dried sawdust was crushed to 0.1–2 mm with a vibratory mill. The samples were placed in a porcelain boat and transferred to a tube furnace for high-temperature carbonization (temperature 1073.15 K, preservation of time 60 min), then cooled to room temperature after carbonization.

The biomass carrier was placed in 10 mL of extractant. The extractant was a mixture of D2EHPA, TBP, and anhydrous ethanol (4:1:5). It was soaked under 100 W ultrasonic equipment for 5 min, then washed with deionized water. The new adsorbent material SICB was obtained.

#### 2.2.2. Adsorption Experiments of Vanadium

The initial vanadium solution was adjusted to the desired pH = 1.0–2.0 using 2 mol/L sulfuric acid or 1 mol/L sodium hydroxide. SICB and the adjusted vanadium solution were mixed in a conical flask at a solid-liquid ratio of 1:(10–40) g·mL and placed in a thermostatic shaker (frequency 200 r/min) for a period of 0.5–24 h for the adsorption experiment. At the end of the adsorption, a solid-liquid separation was carried out and the ion concentration in the solution after adsorption was determined. The concentration of vanadium was analyzed by titration with ferrous ammonium sulfate. The desorbent was mixed with the adsorbed SICB in a liquid to solid ratio of 1:20 g·mL and then placed in a constant temperature oscillator and shaken (frequency 200 r/min) for a desorption test with a desorption time of 0.5–2 h. After desorption, the solid-liquid separation was completed and the ion concentration of the after-desorbed solution was determined.

#### 2.2.3. Analysis Methods

Vanadium concentrations were analyzed by titration with ferrous ammonium sulfate.

The specimen was decomposed by sulfuric acid and phosphoric acid in a 12% (*v*/*v*) to 20% (*v*/*v*) sulfuric acid solution at a temperature below 20 °C. The vanadium, chromium, and possibly oxidizing substances were reduced to low values by the addition of ferrous sulfate solution and then oxidized to pentavalent by potassium permanganate, at which point the chromium was not oxidized. Excess potassium permanganate was destroyed by the decomposition of urea, which did not affect the determination of vanadium. The solution was titrated with a standard solution of ammonium ferrous sulfate using *N*-phenyl ophthalmic acid as an indicator until the solution changed from purplish-red to light green or light yellow-green. The vanadium content was calculated. The concentrations of Fe, Al, Mg, K, and P in the liquids were analyzed by ICP-OES (ICP AES; IRIS Advantage Radial, Thermo Scientific, Waltham, MA, USA). The morphology of the carbonized biomass at different stages and the elemental distribution of the biomass before and after adsorption were observed using scanning electron microscopy (JSM-IT300, Jeol, Tokyo, Japan). The specific surface area of carbonized biomass samples was evaluated by the Barren-Emmett-Teller (Micromeritics) method. The material composition of biomass and carbonized biomass was analyzed using X-ray diffraction (XRD; Bruker D8 Advance, Bremen, Germany). The pH value of the solution was measured using a PHS-3C digital pH meter (INESA Scientific Instruments Co., Ltd., Shanghai, China).

The removal percent (*R*, %) of vanadium (IV) was calculated using the equation:

(1) $$R=\frac{C1-C2}{C1}\times 100\%$$

where (*R*, %) was the removal percent of a concentration of V(IV), and *C*1 and *C*2 were initial and equilibrium concentrations of V(IV) in the solutions (mg·L^−1^) respectively.

## 3. Results and Discussion

### 3.1. Effect of Different Biomass Matrices on the Adsorption Effect

**Figure 1:**
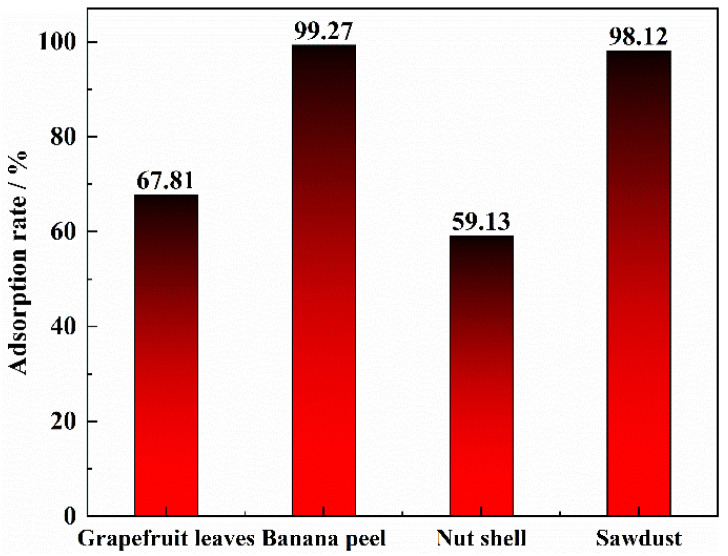
Vanadium adsorption rate of different biomass species (Preparation conditions of biomass species: heat preservation temperature = 1073.15 K; preservation of time = 60 min; impregnation time = 5 min; pH = 1.6; Solid/liquid = 1:20 g·mL; adsorption time = 24 h).

The carriers were prepared from fruit peels, leaves, nut shells, and wood chips at a preparation temperature of 1073.15 K and cooled to room temperature. Di(2-Ethylhexyl) phosphoric acid and tributyl phosphate were impregnated for 60 min. The biomass was adsorbed for 24 h at an initial V(IV) solution concentration of 1.1 g/L, pH 1.6, and material to liquid ratio of 1:20 g·mL. The effect of different types of biomass on vanadium adsorption is shown in Figure 1. The adsorption of vanadium was poor for both grapefruit leaves and fruit shells, with 67.81% and 59.13% adsorption of vanadium. respectively, which may be because grapefruit leaves and fruit shells have fewer pores after carbonization, have a smaller specific surface area, and can be loaded with less extractant. In addition, the adsorption effect of sawdust is better, reaching 98.12%, while the adsorption effect of banana peel is the highest value, reaching 99.18%. The sawdust and banana peel may have more pore structure and larger specific surface area after high-temperature carbonization. However, banana peels are not easily available on a large scale in practical production, whereas sawdust is widely available and easily accessible. Therefore, sawdust was chosen as the best biomass substrate in this experiment.

### 3.2. Effect of Carbonation Temperature and Time on Vanadium Adsorption from Biomass

**Figure 2:**
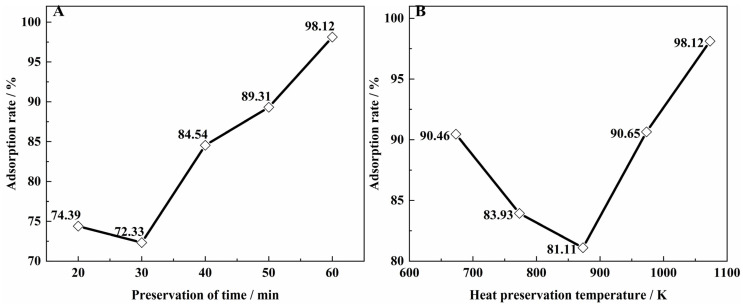
Vanadium adsorption rate of different preparation of biomass, (**A**) is the preservation time and (**B**) is the heat preservation temperature (Preparation conditions of biomass species: biomass species = sawdust; pH = 1.6; impregnation time = 5 min; pH = 1.6; solid/liquid = 1:20 g·mL; adsorption time = 24 h).

Temperature and time affected the structure of carbonized biomass. The adsorption rate of vanadium from biomass prepared at different temperature and time conditions is shown in Figure 2. When the preservation temperature reached 1073.15 K and the preservation time reached 60 min, the adsorption effect was able to reach 98.12%. The carbonized biomass became a porous structure. As time increased, the porosity increased and the structure stabilized. As shown in Figure 2B, the adsorption rate decreased as the temperature increased from 673.15 K to 873.15 K. This may be due to the change in the specific surface area of the biomass during the heating process. The adsorption rate decreased.

### 3.3. Effect of Particle Size of Carbonized Biomass on the Adsorption of Vanadium

**Figure 3:**
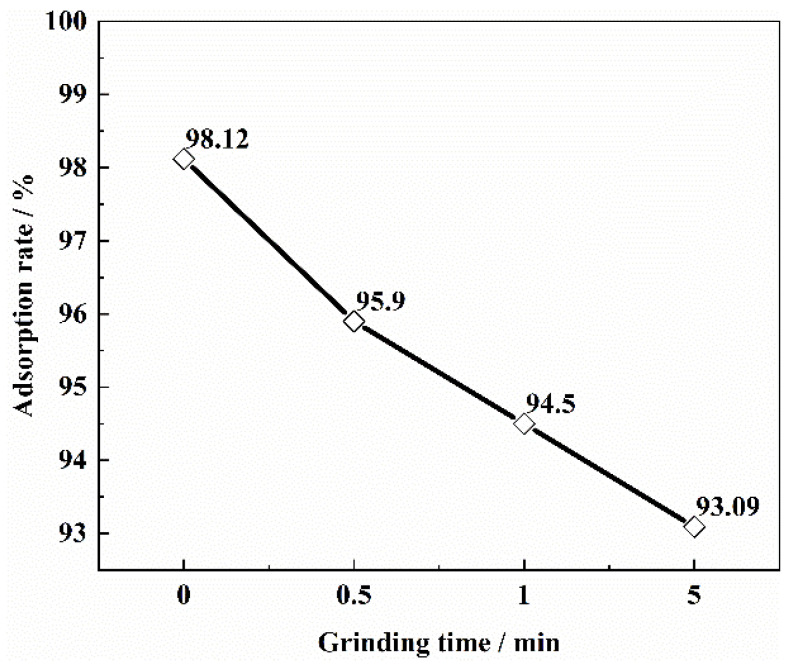
Vanadium adsorption rate of SICB particle size (Preparation conditions of biomass species: biomass species = sawdust; heat preservation temperature = 1073.15 K; preservation of time = 60 min; impregnation time = 5 min; pH = 1.6; solid/liquid = 1:20 g·mL; adsorption time = 24 h).

The effects of different particle sizes on vanadium ion adsorption are shown in Figure 3. As the particle size decreases, the adsorption rate also tends to decrease. The particle size was reduced from 2 mm to 0.180 mm, and the adsorption rate decreased by 4.93%. The decrease in the adsorption rate of vanadium may be due to the following reasons: a decrease in particle size, an increase in specific surface area, and possible destruction of some micro and mesoporous structures resulting in a decrease in the actual specific surface area for adsorption. Therefore, 1–2 mm was used as the optimal biomass particle size in this paper.

### 3.4. Effect of SICB Impregnation Time on Vanadium Adsorption

**Figure 4:**
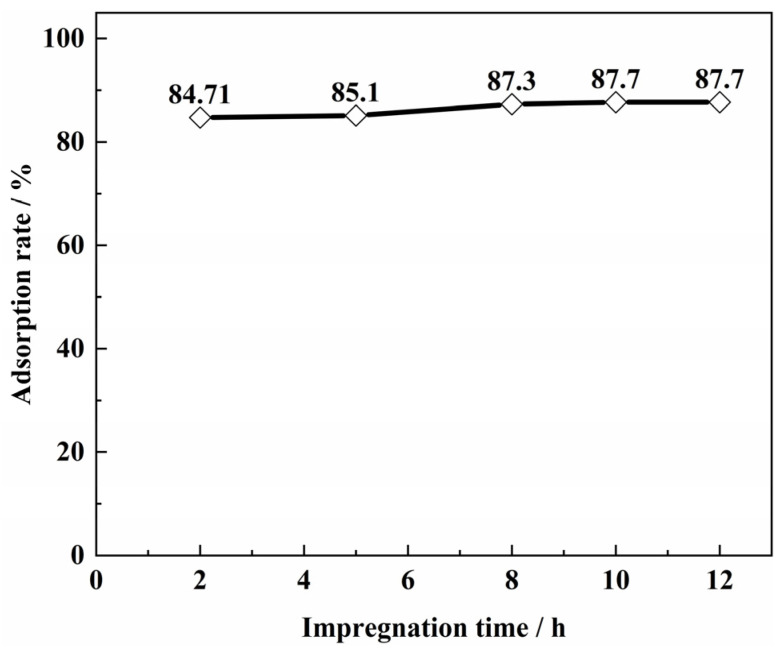
Vanadium adsorption rate of SICB particle size (Preparation conditions of biomass species: biomass species = sawdust; heat preservation temperature = 1073.15 K; preservation of time = 60 min; pH = 1.6; solid/liquid = 1:20 g·mL; adsorption time = 12 h).

The effect of impregnation time on vanadium ion adsorption is shown in Figure 4. The adsorption rate tended to increase with the increase of impregnation time. The weight of SICB was measured. It was found that the increase in impregnation time did not increase the content of the loading solvent. The increase in impregnation time may result in better dispersion of the solvent on the specific surface area of SICB. However, it was impregnated for 5 min and adsorbed for 24 h to meet the experimental requirements. Therefore, 5 min was chosen as the optimal experimental impregnation time in this study [15].

### 3.5. Characterization of Carbonized Biomass and SICB

#### 3.5.1. Surface Topography of Carbonized Biomass and SICB

**Figure 5:**
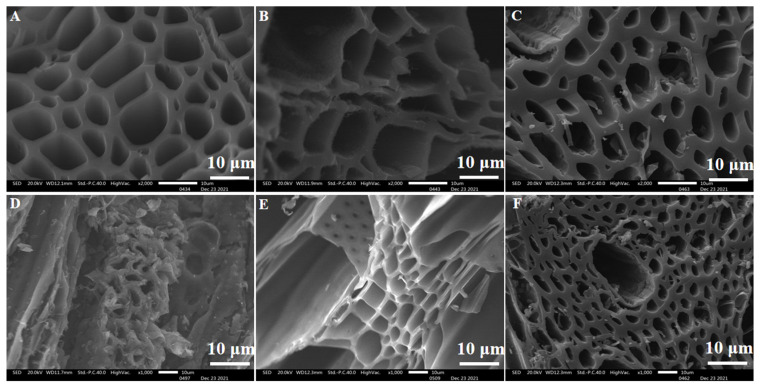
SEM images of heat preservation temperature and preservation of time: (**A**) CB400, (**B**) CB600, (**C**) CB800, (**D**) CB20, (**E**) CB40, (**F**) CB60.

The carbonized biomass obtained by a heat preservation temperature of 673.15 K–1073.15 K are named CB400, CB600, and CB800, respectively, and Figure 5 shows the SEM image of them. The carbonized biomass obtained by the preservation of time at 20 min to 60 min are named CB20, CB40, and CB60, respectively, and Figure 5 shows the SEM image of them. The specific surface of CB600 is not very smooth. Some materials can be seen growing on the surface in Figure 5B. This may be because some volatiles started to evaporate between 773.15 and 873.15. These volatiles were not completely volatilized under temperature control because the adsorption rate first decreased and then increased with the increase in temperature. It can be seen that with the increase in the carbonization temperature, the pore structure of CB improves noticeably, indicating that CB has been prepared successfully. The structure of the CBs has not changed. They were composed of a layered structure in a certain direction with some holes. These pores were arranged irregularly and widely distributed, which was one of the reasons for the large specific surface area of biomass. This was also one of the reasons for the large adsorption capacity [27]. When vanadium ions reach the surface of the CB, some of them are adsorbed on the outer surface, while the adsorbed vanadium ions may diffuse to the interior due to the pores of the CB. It is due to this behavior that the pore structure of CB improves significantly when the carbonization temperature is increased. The adsorption effect increases as well.

#### 3.5.2. Specific Surface Area Test of Biomass and CB

As shown in Table 1, as the carbonization temperature continuously increased, the specific surface area, the t-plot micropore area, and the t-plot external surface area of CB continuously increased. When the carbonization temperature reached 1073.15 K, the specific surface area, the t-plot micropore area, and the t-plot external surface area of CB exceed that of the biomass. The carbonization of biomass produces mesopores and micropores.

### 3.6. Effect of Adsorption Process on the Adsorbed Vanadium of SICB

**Figure 6:**
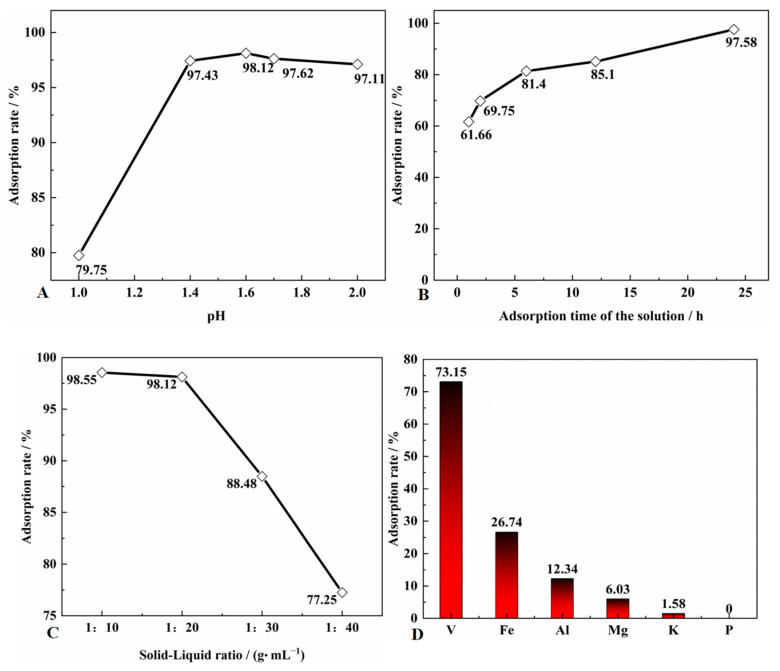
Vanadium adsorption rate of adsorption process under different conditions (**A**) is the adsorption rate without pH, (**B**) is the adsorption rate at different times, (**C**) is the adsorption rate at different liquid to solid ratios and (**D**) is the adsorption rate for different elements (Preparation conditions of biomass species: biomass species = sawdust; heat preservation temperature = 1073.15 k; preservation of time = 60 min; impregnation Time = 5 min; pH = 1.5/1.6/1.6; solid/Liquid = 1:20 g·mL; adsorption Time = 24 h).

As shown in Figure 6A, the adsorption rate of vanadium increases significantly with increasing pH in the range of pH = 1.0–1.4, and then decreases as pH continues to increase, with the highest adsorption rate of 98.12% at pH = 1.6. The change in pH represents the change of hydrogen ions. When there is a large number of hydrogen ions in the solution, D2EHPA will not dissociate the hydrogen ions, so the adsorption effect is not good. When pH increases, the vanadium ion morphological transformation cannot be adsorbed. Figure 6B shows that the adsorption rate of vanadium by SICB increased with time, up to 97.58% within 24 h. The adsorption process is a slow mass transfer process that requires some time to accumulate before saturation is reached. Figure 6C shows the effect of different solution ratios on the adsorption rate of vanadium, which decreased by 0.43% when the vanadium solution was doubled. When the vanadium solution was doubled, the adsorption rate decreased by 20.87%, so 1:20 was chosen as the optimum liquid-to-solid ratio in this paper. As the vanadium ion concentration decreases, the equilibrium inside and outside the extraction interface is more easily balanced, so fewer vanadium ions are adsorbed. From Figure 6D, it can be seen that the adsorption selectivity of SICBs for V, Fe, Al, Mg, K, and P at pH = 1.6 was in the order of Fe^3+^, V^4+^, Al, Fe^2+^, Mg, K, and P [28]. In our experiments, the high adsorption rate of Fe may be due to the partial oxidation of Fe^2+^ to Fe^3+^, so the adsorption rate of Fe was higher than that of other ions. The decrease in vanadium ion adsorption rate was due to the decrease in effective adsorption capacity, leading to the adsorption of other ions.

### 3.7. Element Distribution of SICB before and after Adsorption

**Figure 7:**
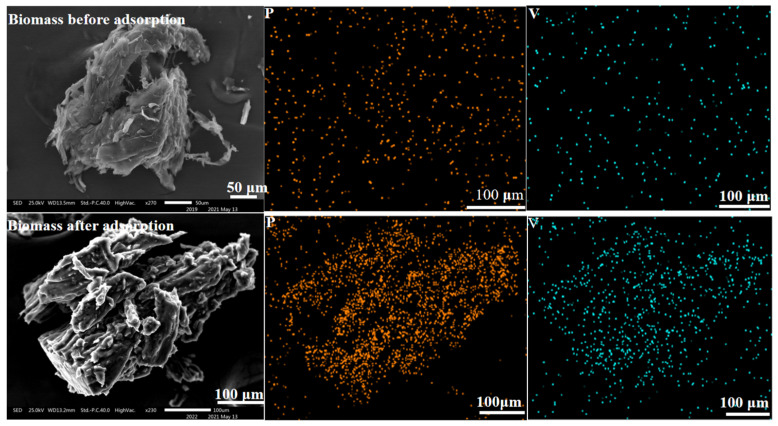
Comparison of elements **before** and **after** biomass adsorption.

SEM images of the biomass before and after adsorption are shown in Figure 7. By surface scanning, the carbonized biomass was found to be free of oxygen and phosphorus, while SICB contained oxygen and phosphorus. The impregnation method was a successful solvent-loading method. The presence of vanadium also explained the successful adsorption of vanadium (IV) ions from a microscopic point of view.

### 3.8. Effect of Desorption on Vanadium Desorption Agent

**Figure 8:**
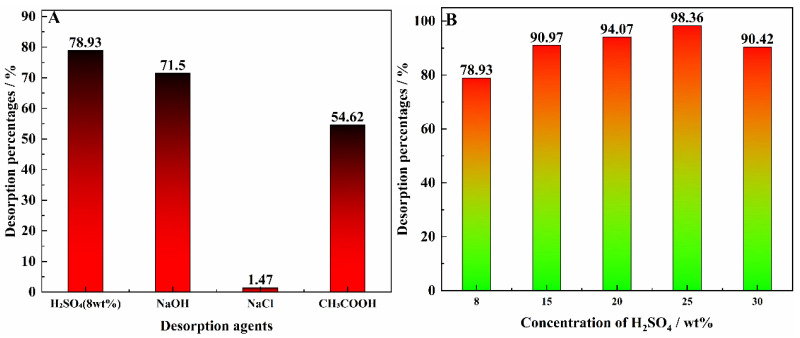
Effect of the desorbent on the vanadium desorption agent percentage. (**A**) is the desorption rate for different types of desorbent and (**B**) is the desorption rate for different concentrations of desorbent.

The experimental results are shown in Figure 8A. All of the reverse extract solutions had a solubility of 1 mol/L, except sulfuric acid. The results plot showed that NaCl exhibited almost no desorption agent ability. Acidic resolvers, such as CH_3_COOH, are also insufficient as desorbents. A desorption agent percentage of 71.50% could be obtained by 1 mol/L NaOH. However, neither of them meets the expected desorption agent requirements. The desorption agent percentage of the mixed solution of 8 wt% H_2_SO_4_ was as high as 78.93% [29].

After discussing the use of H_2_SO_4_ as a desorption agent, the concentration of H_2_SO_4_ in the desorbent system was further considered. The experimental results of the effect of H_2_SO_4_ concentration on vanadium desorbent are shown in Figure 8B. As can be seen from Figure 8B, the percentage of vanadium desorption increased from 78.93% to 98.36% as the concentration of H_2_SO_4_ increased from 8 wt% to 25 wt%. At concentrations above 25 wt%, the percentage of vanadium desorption decreases to about 90%. Therefore, to achieve maximum desorbent effect, the resolver was determined to be a mixture of 25 wt% H_2_SO_4_.

### 3.9. Modeling of Adsorption of V(IV) on SICB

#### 3.9.1. Adsorption Kinetics of SICB on V(IV)

At constant temperature, the adsorption rate of SICBs showed a functional relationship with increasing adsorption time. Experiments were performed at 303 K to determine the adsorption rate of SICB on V(IV) ions, where 1.1 g/L V(IV) vanadium solution was used. These results were examined using pseudo-first- and pseudo-second-order reference studies [30,31], achieving maximum adsorption capacity and quantitative adsorption within 24 h at 303 K. Pseudo-first-order and pseudo-second-order models were used to simulate the V(IV) adsorption kinetics on SICB. The kinetics were obtained by fitting the experimental data with the mentioned models and are summarized in Table 2.

The expressions of the quasi-first-order and quasi-second-order kinetic equations are Equations (2) and (3).

(2) $$\ln(Q_{e}-Q_{t})=\ln Q_{t}-k_{1}t$$

(3) $$\frac{t}{Q_{t}}=\frac{1}{k_{2}Q_{e}^{2}}+\frac{t}{Q_{e}}$$

where *Q_e_* and *Q_t_* were the amounts of adsorbed V (mg/g) at equilibrium and time *t* (h).

**Figure 9:**
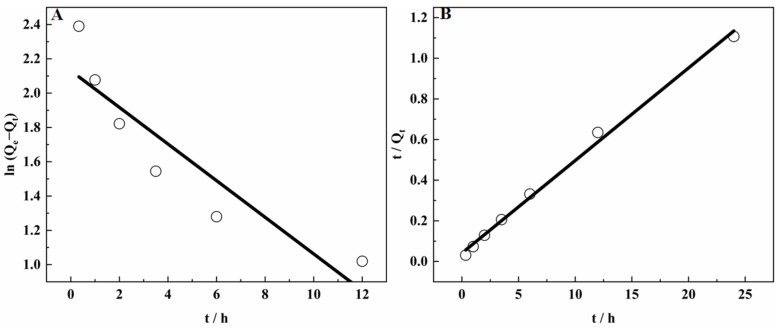
Effect of the adsorption time on the amount of V(IV) adsorbed on SICB at 303 K: (**A**) is pseudo-first-order plot and (**B**) is pseudo-second-order plot.

As shown in Figure 9, the pseudo-second-order model was used to simulate V(IV) adsorption kinetics on SICB. The pseudo-first-order model was better fitted, based on the following numerical regression coefficients (R^2^). This suggested that the adsorption process of SICB was more in line with the pseudo-second-order kinetic principle. According to the overall process described by the pseudo-second-order kinetic equation, the initial concentration of the solution had a strong influence on the adsorption rate, and the adsorption process was dominated by chemisorption [32,33].

#### 3.9.2. Adsorption Process of V(IV) in SICB

The carbonized biomass itself is physisorbed, but the saturation capacity for physical adsorption is low and the adsorption is not selective. The vanadium shale has a low vanadium content and the leachate contains many impurity ions. Some of the impurity ions are in higher concentration than vanadium ions, so if only carbonized biomass is used, adsorption of vanadium ions is poor. When using impregnated carbonized biomass, it has its own specific adsorption sites for vanadium ions. Therefore, it has a better selectivity for vanadium ions. In the process of vanadium ion adsorption on impregnated carbonized biomass, the carbonized biomass not only acts as a carrier, but also has a synergistic effect of physical adsorption [18,34].

The impregnation process can be divided into two categories. One relied on the principle of suspension polymerization, where the extraction agent could be added directly to the process of raw material production, and the reaction occurred together [35]. The second type was to dissolve the extractant in common inert diluents (anhydrous ethanol, kerosene, petroleum ether, and other organic solvents), mix the carrier with the extracting solvent, and load the carrier using physical activities such as hydrophobic interaction between the alkane chain of the extractant and the hydrophobic group on the carrier skeleton [36].

Because the majority of vanadium ions present in the pH 1–2 medium were in the form of the vanadium oxide ion VO^2+^ [1], the hydrogen bond in the D2EHPA dimer was partially broken, prompting the D2EHPA of the monomer to be able to undergo an adsorption reaction with vanadium ions. During the adsorption process, there was a cation exchange reaction between the vanadyl and vinylidene ions and the hydrogen ion of the P-OH group in D2EHPA. The phosphoryl group (P = O) was involved in the coordination reaction of the vanadyl and vinylidene ions. Both of them acted together to realize the adsorption reaction of SICB with vanadium ions [37].

The main role of TBP was to reduce the time to adsorption equilibrium of D2EHPA [12].

**Figure 10:**
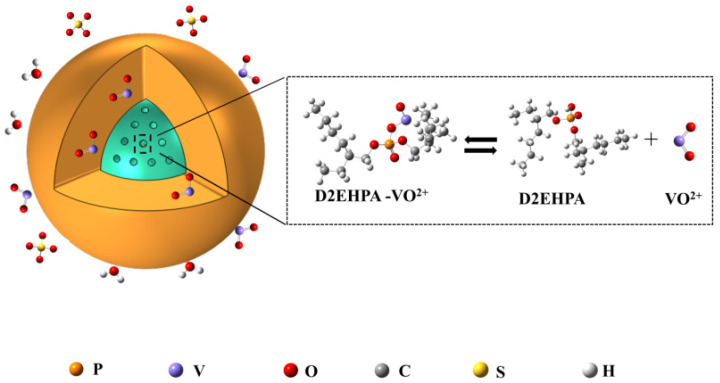
The mechanism of V(IV) adsorption on SICB.

The solvent-impregnated biomass was assumed to be an extraction solvent uniformly dispersed in the carrier biomass. The carrier acted only as a loaded extraction solvent. The main part of the chemical reaction that occurred still involved the extractant and metal ions. In other words, SICB reacted not only with vanadium ions on the surface of the biomass, but also with the extractant inside the biomass, and there was a liquid-solid mass transfer process. According to the “dynamic boundary model” [38], the adsorption process can be divided into three continuously controlled processes:

Vanadium ions in the solution diffuse across a liquid film present on the surface of SICB to the surface of the biomass, called liquid film diffusion movement;

Vanadium ions move inside SICB and reach the vicinity of extractant groups of SICB, called particle diffusion movement;

Metal ions undergo a reverse chemical exchange reaction with extractant groups at the interface of the interior of SICB [39]. The process of vanadium ion adsorption by SICB is shown in Figure 10.

In the initial stage of adsorption, a large number of vanadium ions were present in the solution around SICB, and a large concentration gradient was generated between the solution and the surface of SICB, which accelerated the diffusion of vanadium ions to the surface of SICB; as the exothermic adsorption reaction continued to advance, vanadium ions entered the interior of SICB and chemically reacted with the extractant, and the vanadium ion concentration in the solution gradually decreased. The diffusion resistance became larger, resulting in the adsorption rate becoming slower. At the later stage of adsorption, the vanadium ion concentration became lower and lower. The diffusion resistance became greater and the diffusion rate became smaller, and the adsorption reaction finally reached equilibrium. To reflect the adsorption mechanism of vanadium by SICB, liquid film diffusion, particle diffusion, and chemical reaction control equations were used to fit the adsorption process, respectively, where the slowest step was the velocity control step [40]. The liquid film diffusion, particle diffusion, and chemical reaction control equations are expressed as (4)–(6).

(4) $$F=\frac{3C_{o}K_{f}}{aQ_{r}}t=kt$$

(5) $$1-3{\left(1-F\right)}^{\frac{2}{3}}+2(1-F)=\left[\frac{6DCo}{aQ_{r}^{2}}\right]t=kt$$

(6) $$1-{\left(1-F\right)}^{\frac{1}{3}}=(\frac{k_{c}C_{o}}{r})t=kt$$

In the equation, *Q_e_* was the equilibrium concentration of vanadium ions in solution (mg/L) and *Q_t_* was the equilibrium adsorption of V(IV) by D2EHPA and TBP impregnated biomass (mg/g). In Equation (4) where *F* (*F* = *Q_t_*/*Q_e_*) was the exchange degree and *Co* was the initial concentration of vanadium ions in solution (mol/L), *K_f_* was the liquid film diffusion control coefficient (cm/m); *a* was the stoichiometric unit; *t* was the reaction time, min; and *K* was the apparent rate number in cm^4^/(mol·s). In Equation (5), the effective diffusion coefficient was *D* SICB. In Equation (6), *r* was the radius of SICB particles, m; *K_c_* was the chemical reaction control rate constant in cm^4^/(mol·s).

**Figure 11:**
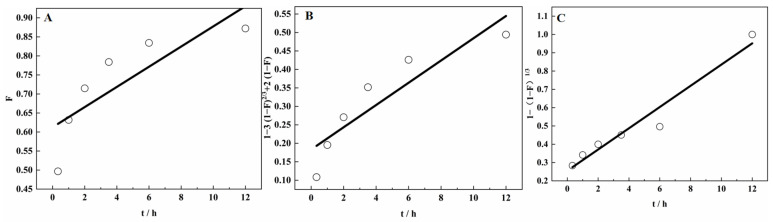
Fitted curves for velocity control steps in the adsorption exchange process, (**A**) is liquid film diffusion fitted curve, (**B**) is particle diffusion fitted curve and (**C**) is chemical reaction control fitted curve.

The experimental data were linearly fitted to t according to *F*, [1 − 3(1 − *F*)^2/3^ + 2(1 − F)] and [1 − (1 − *F*)^1/3^], and the speed control step of the adsorption exchange was judged according to the correlation of the linear fit. The fitted curve is shown in Figure 11A–C.

As can be seen from Table 3, the correlation coefficient of the fitted curve for the chemical reaction control of V(IV) is 0.95336, which has high linearity, with the additional benefit of sufficient mixing, fast diffusion, and the large specific surface area of the organisms. Therefore, the rate control step of SICB for the V(IV) adsorption process was a chemical reaction.

#### 3.9.3. Adsorption Isotherm of V(IV) on SICB

**Figure 12:**
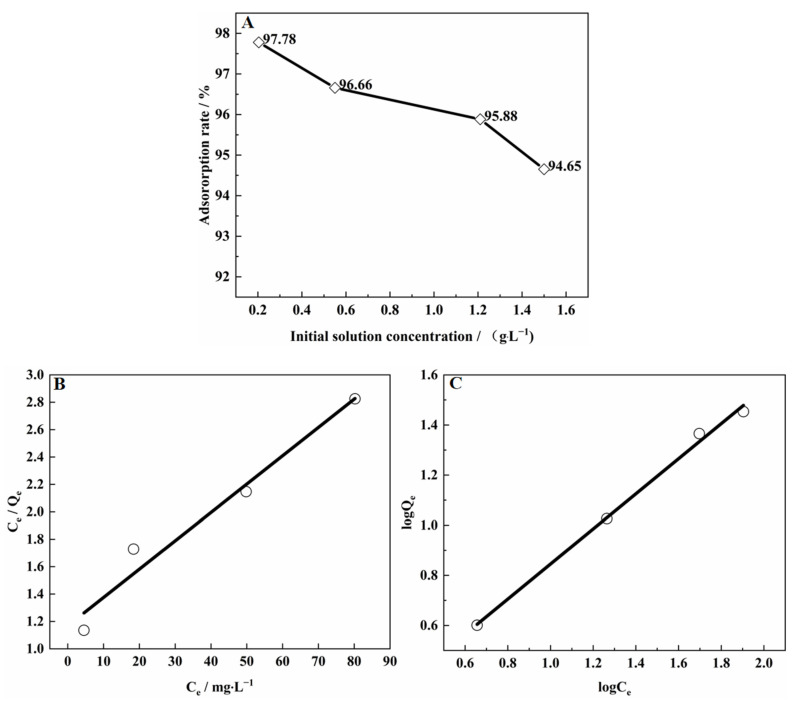
V(IV) adsorption rate of different initial vanadium concentrations, (**A**) is the adsorption curve for different initial concentrations, (**B**) is Langmuir and (**C**) is Freundlich.

At a temperature of 303 k, the adsorption experiment was carried out with solutions of different initial vanadium concentrations of 0.045–1.5 g/L^−1^, and the results are shown in Figure 12A. The isotherms for adsorption of V(IV) on the SICB, from low to high V(IV) concentration solutions at constant temperature shaker (303 K), are shown in Figure 12B,C. The isotherms were obtained by fitting the experimental data with the mentioned models and are summarized in Table 2 [41]. The expressions of the Langmuir and Freundlich thermodynamic equations are Equations (7) and (8).

(7) $$\frac{C_{e}}{Q_{e}}=\frac{C_{e}}{Q_{m}}+\frac{K_{1}}{Q_{m}}$$

(8) $$\log Q_{e}=\log K_{F}+\frac{\log C_{e}}{n}$$

In the equation, *C_e_* was the equilibrium concentration of vanadium ions in solution (mg/L); *Q_e_* was the equilibrium adsorption of V(IV) by D2EHPA and TBP impregnated biomass (mg/g); *Q_m_* was the theoretical saturation adsorption of V(IV) by D2EHPA and TBP impregnated biomass during adsorption (mg/g); *K_L_* was the Langmuir isothermal adsorption equilibrium constant; and *n* and *K_F_* were Freundlich isothermal adsorption equation constants.

By comparing the R^2^ of both Langmuir model parameters and Freundlich model parameters, it was deduced that the better match for vanadium adsorption through biomass was the Freundlich model. The various parameters obtained from the Freundlich plot are given in Table 4. *n* was a measure of the intensity of adsorption. *n* < 1 indicated normal adsorption, whereas 1 < *n* < 10 indicated a favorable adsorption process [42]. Therefore, from the value of *n* = 1.43, the impregnation of biomass at the initial concentration tested was considered a reasonable adsorption process.

The maximum adsorption capacity was a theoretical value derived from different initial concentrations, which was 48.34 mg/g. The saturation capacities of the three extraction methods are shown in Table 5. Compared with D2EHPA and TBP-impregnated resin [11], the saturated capacity increases by 61.40% (48.34 mg/g versus 29.95 mg/g).

## 4. Conclusions

This study shows that sawdust biomass can adsorb the material matrix and produce an effective V(IV) biosorbent. Sawdust was the most suitable biomass source at a carbonization temperature of 1073.15 K and a carbonization time of 1 h. The optimal conditions for vanadium extraction from biomass impregnated with di-(2-Ethylhexyl) phosphoric acid- tributyl phosphate for 10 min at a particle size of 1–2 mm were an initial pure vanadium solution pH of 1.6, a sorption time of 24 h, and a solid/liquid ratio of 1:20 g·mL. This wood biosorbent adsorbed up to 98.12% of V(IV). The uptake of V(IV) by SICBs at 303 K followed the Freundlich isotherm in the simulated liquid concentration range (1–1.5 g/L) after leaching, and the adsorption kinetics could be described by pseudo-second-order kinetics. Through SEM-EDS analysis, it was found that the impregnated biomass contained an organic phase, which could act as a sorbent for V(IV) ions. More than 98% of the vanadium in the loaded organic phase can be quantitatively desorbed with a 25 wt% sulfuric acid solution. Compared with D2EHPA and TBP-impregnated resin, the saturated capacity increased by 61.40%. Experimental studies with SICB have shown that under certain conditions, it can be used as a mechanism for purification and enrichment in a process to recover vanadium from vanadium shale leach liquor.

## Figures and Tables

**Table 1:** The specific surface area before and after carbonization of biomass.

Category	BET Surface Area m^2^/g	Langmuir Surface Area m^2^/g	t-Plot Micropore Area m^2^/g
Biomass	0.76	0.80	0.76
CB	148.88	151.38	124.92

**Table 2:** Kinetic parameters of V(IV) adsorption on SICB.

Kinetic Model	Pseudo-First-Order			Pseudo-First-Order		
	R^2^	Q	K	R^2^	Q	K_2_
	0.83225	135.114	0.1068	0.9954	21.990	0.0502
	y = 2.1307 − 0.10679 x			y = 0.0456 x + 0.04114		

**Table 3:** Fitting parameters for the velocity control step in the adsorption exchange process.

Liquid Film Diffusion Fitted Curve		Particle Diffusion Fitted Curve		Chemical Reaction Control Fitted Curve	
R^2^	0.67772	R^2^	0.82137	R^2^	0.95226
y = 0.61261 + 0.02649 x		y = 0.18305 + 0.03013 x		y = 0.25508 + 0.05802 x	

**Table 4:** Fitting parameters of the adsorption isotherm model.

Isotherm Model	Langmuir			Freundlich		
	Q	K_L_	R^2^	*n*	K_F_	R^2^
	48.34	0.024	0.966	1.43	1.40	0.9962
	y = 0.02068 + 1.16817 x			y = 0.014512 + 0.70009 x		

**Table 5:** Saturated capacity for three extraction methods.

Category	Saturated Capacity/mg·g^−1^
D2EHPA and TBP	24.21
D2EHPA and TBP impregnated resin	29.95 [11]
D2EHPA and TBP impregnated biomass	48.34

## Data Availability

Not applicable.
